# The Influence of Aerobic Power on Repeated Anaerobic Exercise in Junior Soccer Players

**DOI:** 10.2478/v10078-011-0023-z

**Published:** 2011-07-04

**Authors:** Lukas Cipryan, Vojtech Gajda

**Affiliations:** 1 Centre for Diagnostics of Human Movement, Department of Physical Education, Ostrava University

**Keywords:** intermittent activity, maximal oxygen uptake, treadmill test, shuttle run, physical efficiency

## Abstract

The main purpose of the present study is to investigate the relationship between anaerobic power achieved in repeated anaerobic exercise and aerobic power. The study group consisted of 40 soccer players (age 17.3 ± 1.36 years). All participants performed 3 tests: a running-based anaerobic sprint test (RAST), a graded treadmill test (GXT), and a multistage fitness test (20mPST). A statistically significant correlation was found among peak power in the GXT and the maximum (r = 0.365, p=0.02), minimum (r=0.334, p=0.035) and average (r=0.401, p=0.01) power in the RAST. No relationships were found between VO_2_max obtained from both aerobic tests and any performance indices in the RAST. A statistically significant correlation was found between the VO_2_max obtained from the spiroergometry examination (GXT) and the calculated VO_2_max of 20mPST (r=0.382, p=0.015). In conclusion, the level of VO_2_max does not influence the performance indices in the RAST in elite junior soccer players. It is possible that the modification of anaerobic test protocol or a more heterogeneous study group would influence the results. The estimation of the VO_2_max in the 20mPST is too inaccurate and should not replace the laboratory spiroergometry examination.

## Introduction

Soccer is the most popular sport in the world and is played regardless of such factors as age, sex, race, fitness level or sport performance. It depends on a number of factors such as technical and tactical skills, mental readiness and physiological factors (Hoff et al., 2002; Stølen et al., 2005). Therefore, the development and a high level of physical capacity cannot be the only single indicator of a successful player, but represent the fundamental prerequisite of game performance (Chamari et al., 2004). Field-based team sports are primarily characterized by repeated maximal exercise lasting approximately 2–4 seconds (10–20 m distance) with an approximately 90 s recovery (Bangsbo et al., 1991; Spencer et al., 2005). The total distance during an elite soccer match is approximately 10–13 km. Most of this distance, however, is covered by walking and low-intensity running, which demand a limited energy production (Stølen et al., 2005; Bangsbo et al., 2006). The total distance covered and running intensity during competitive matches in young soccer players can differ between age groups. In the study of Brazilian youth players, the running intensity of the U15 (under-15 years) players (118 m.min^−1^) was significantly greater then U17 (108 m.min^−1^) and U20 (109 m.min^−1^) (Pereira Da Silva et al., 2007).

The energy resynthesis for muscle contraction in soccer mostly occurs through the aerobic pathway, even when the movement consists of repeated and very intensive short sprints. It is obvious that the relative contribution of anaerobic glycogenolysis is reduced during the performance of subsequent sprints, which is partially explained by an increase in aerobic metabolism (Gaitanos et al., 1993; Bogdanis et al., 1996; Parolin et al., 1999; Spencer et al., 2005). Aerobic endurance performance is influenced by three important elements: maximal oxygen uptake (VO_2_max), anaerobic threshold and work economy (Hoff et al., 2002). The relationship, however, between the VO_2_max and anaerobic power have been inconsistent. Althought some authors did not confirm this relationship to be sufficiently close (Bell et al., 1997; Wadley & Le Rossignol, 1998; Hoffman et al., 1999; Aziz et al., 2000), others suggested that VO_2_max is a prerequisite for intermittent activities (Tomlin & Wenger, 2001, 2002; Bishop, 2004). Consequently, according to Aziz et al. (2000), improving aerobic fitness further should only be expected to contribute marginally to improved repeated sprint performance for team game players. The verification of this fact is very important for creating an effective training program.

Physical capacity diagnostics is a necessary part of the professionally conducted training process. It provides important feedback regarding the current fitness level, its increasing or decreasing trend and the readiness for sport performance. This information can be used for the training program adjustment or the players’ selection. Due to the character of the exercise load in field-based team sports, it is necessary to find appropriate exercise tests for repeated maximal exercise, which demand sufficiently developed anaerobic capabilities. Repeated sprint ability (RSA) is the ability to perform repeated sprints with a short recovery between sprint bouts. The repeated sprint bout consists of at least three sprints with a mean recovery time between sprints of less than 21 seconds (Spencer et al., 2004).

The main purpose of the present study was to investigate the relationship between the anaerobic power achieved in repeated anaerobic exercise, which is characteristic for movement pattern in soccer, and aerobic power (VO_2_max). Thus, we tested the hypothesis that VO_2_max positively influences the performance indices of the repeated anaerobic exercise test in a group of elite junior soccer players. An additional aim of the study was to examine the differences between VO_2_max predicted from indirect measurement by means of a multistage fitness test with a direct measurement of VO_2_max during treadmill running.

## Material and methods

### Participants

Forty junior soccer players (age 17.3 ± 1.36 years; weight 71.3 ± 5.27 kg; height 179.8 ± 5.30 cm; body fat 13.9 ± 2.21 %) volunteered to participate in this study. The participants were informed of the study requirements, benefits and risks. All the participants were male players participating in an elite junior soccer league. They have trained at least for ten years, five to six days per week. The study was performed in the middle of the soccer season, when the league was halted for approximately 45 days. Most training sessions at this time of year are devoted to conditioning first and then, specific tactical drills and game skills with the use of balls. The protocol of this study was in accordance with the guidelines of the Ethical Committee of Ostrava University and Declaration of Helsinki.

### Procedure

The participants performed three tests, separated by at least 48 hours from one other. The tests included the running-based anaerobic sprint test (RAST), the graded treadmill test (GXT) and the multistage fitness test (20mPST), respectively. The participants were asked to refrain from any form of intense physical exercise for the 24-hour period before each testing session. The RAST and 20mPST were performed in the sports hall and the GXT in the laboratory for diagnostics of human movement. None of the participants was taking any food supplements. They were also instructed to not eat for at least 2 hours prior to each test. Before each test, the participants performed an individual 10–15 minute warm up.

### Performance Tests

*Graded Treadmill Test (GXT).* A continuous graded treadmill test was used in order to measure maximal aerobic power (VO_2_max), following the Harbor protocol (Wassermann et al., 2005), which was modified in order for the total time of each test to last 8–12 minutes (Astrand et al., 2003). The test consisted of 3 minutes of a warm-up, followed by constant speed and 2 % inclination increments every minute until the participants reached volitional exhaustion. All participants had the same exercise load throughout the test. The test was stopped when a participant was unable to continue running at the actual velocity and slope. Strong verbal encouragement was provided to each participant as they came to the end of the GXT. The test was performed on a Lode Valiant motor-driven treadmill (Groningen, Nederland).

The expired air was continuously monitored for an analysis of O_2_ and CO_2_ concentrations during the GXT by using a breath by breath system (ZAN 600 Ergo; Oberthulba, Germany). Prior to each test, the gas analyzers were calibrated using ambient air with a gas mixture of known O_2_ and CO_2_ concentrations and the ventilatory membrane calibrated with a 3 L syringe in accordance with the manufacturer′s instructions. The ambient conditions were automatically recorded by ZAN 600 Ergo and maintained by air-condition between 20–23° C. The heart rate (HR) was continuously monitored with a chest belt transmission (Polar Electro, Oy, Finland).

Ventilatory data were averaged over 30 second intervals. It was stated that the participants had reached their VO_2_max, when at least 2 of the following criteria were met: (1) a plateau in the VO_2_ despite the increasing running load, (2) a final respiratory exchange ratio (RER) higher than 1.10; (3) an attainment of 95 % of the age-predicted maximal heart rate. The mean values of VO_2_max along with the absolute and relative values of maximal power are shown in Table 1.

*Multistage Fitness Test.* Aerobic power was also evaluated by means of a maximal multistage 20-meter shuttle run test (20-m progressive sprint test, 20mPST). This field test predicts aerobic power (VO_2_max) and has been found to be a sufficiently valid and reliable indicator (Léger & Lambert, 1982). The test consisted of shuttle running at increasing speeds between two cones placed 20 m apart. The pace of the test was dictated by beeps recorded on a CD. The time interval between the emitting tones decreased after each minute. The participants were instructed to always place a foot on or behind the 20 m mark. Each participant was required to be at the opposite end of the 20 m track by the time the next beep sounds. The test was terminated when the participant was unable to reach the 20 m mark twice in succession. The test score achieved was the level and number of shuttles completed immediately prior to the beep on which the participant was eliminated. The VO_2_max was calculated based on the score achieved on the test with the following formula employed (Reiman & Manske, 2009):

$$\begin{array}{c} {\text{VO}}_{2}\text{max}=3.46\times [1\times \text{Level}+(\text{Shuttles}/[\text{Level}\times \\ 4325+7.0048])]+12.2 \end{array}$$

*Repeated Sprint Exercise.* The running-based anaerobic sprint test (RAST) (Mackenzie, 2005) was used in this study. RAST consisted of six 35 m sprints separated by a 10 s recovery. The participants were encouraged to perform each sprint as fast as possible. During the recovery phase, the participant assumed the ready position and awaited the start of the countdown. All sprints began from the standing position. The test was performed in a sports hall. Electronic photocells (EGMedical, Brno, Czech Republic) were used for the sprint time measurement with an accuracy of 0.001 s. The height of the photocell was adjusted in accordance with the height of the participant′s hip. The measured time began when the participant crossed the cell′s beam. The recorded time for each sprint was taken to the nearest hundredth of a second.

The following variables were calculated from the sprint time data (see Table 3): (1) maximum power (W) – the highest value, (2) minimum power (W) – the lowest value, (3) average power (W) – the sum of all six values divided by 6, (4) fatigue index (W.s^−1^) = (maximum power – minimum power) divided by the total time for the six sprints. The power output for each sprint was determined using the following equation:

$$\mathrm{Power}=\mathrm{Weight}\times {\mathrm{Distance}}^{2}÷{\mathrm{Time}}^{3}$$

The body mass and body fat (%) of each participant was measured by Tanita BC 418 MA (Tokyo, Japan). The participants were instructed to not drink for at least 2 hours prior to each bioelectrical impedance measurement.

### Statistical Analysis

All values are reported as mean and standard deviation (SD). The normality distribution of the data was checked with the Shapiro-Wilk test. Pearson product moment correlations were used to assess the relationships between the RAST variables and VO_2_max, and between the GXT and 20mPST VO_2_max values. A paired Student′s t-test was used in order to compare differences between VO_2_max values obtained from GXT and the 20mPST. In addition, the methods of Bland and Altman (2010) were used to assess similarities between these two VO_2_max calculations. The level of significance was set at *p* < 0.05. All statistical procedures were carried out using the PASW Statistics 18 Software.

## Results

The results of the GXT and the 20mPST are summarized in Table 1. The performance indices of the RAST are summarized in Table 3.

**Figure 1:**
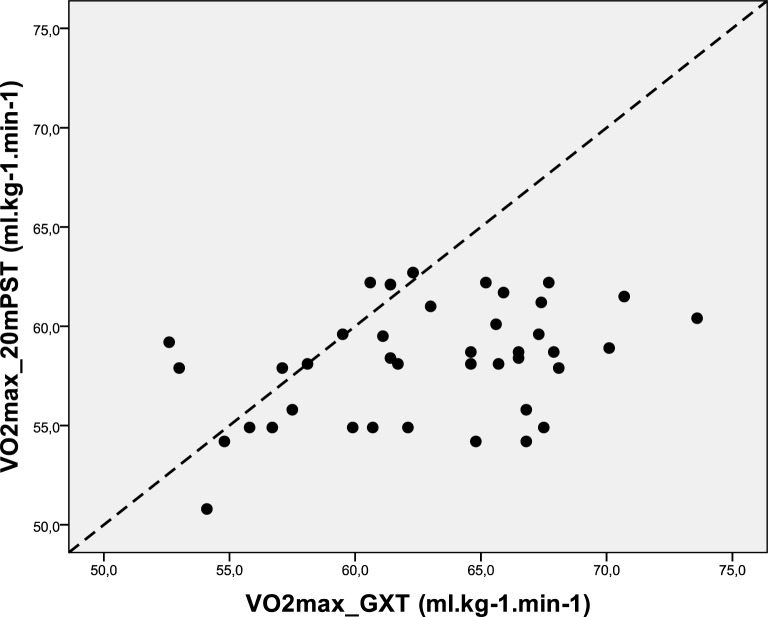
Scatter plot of GXT and 20mPST VO_2_max (with line of equality superimposed)

It is apparent from Figure 1 that there is a low relationship between the VO_2_max in GXT and 20mPST. There is evidence that the VO_2_max from the 20mPST tends to underestimate the VO_2_max from the GXT by between 3.19 and 6.27 ml.kg^−1^.min^−1^ on average (Table 2). A statistically significant correlation was found between VO_2_max obtained from the spiroergometry examination (GXT) and the calculated VO_2_max of the 20mPST *(r = 0.382, p = 0.015, r^2^* *= 0.146).*

**Figure 2:**
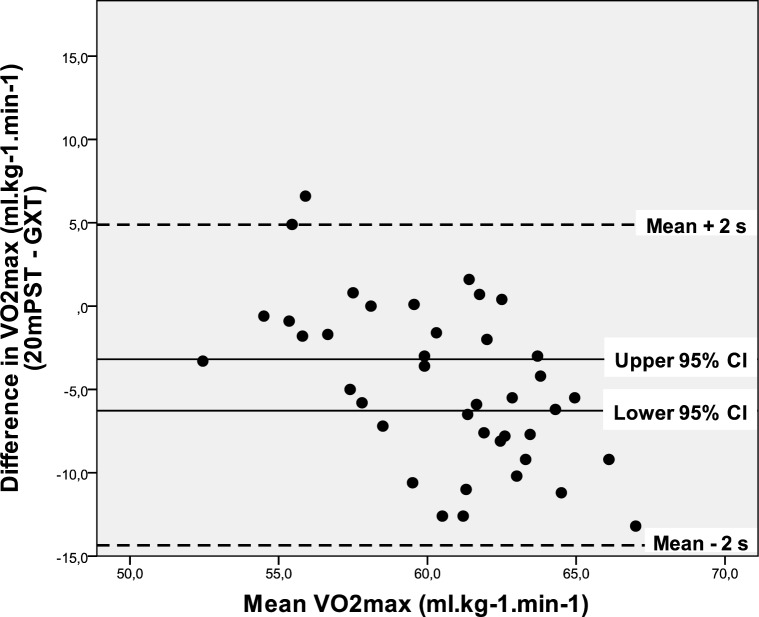
Bland-Altman plot of difference against mean for VO_2_max data

Using the output from Table 2, the approximate 95% limits of agreement (mean difference ± 2 s) are −14.35 to 4.89 ml.kg^−1^.min^−1^. Therefore, it is expected that 95 % of this specific population will have differences between their 20mPST and GXT measurements in this range (Figure 2).

The correlations among the results of the anaerobic (RAST) and aerobic (GXT, 20mPST) tests are summarized in Table 4. Statistically significant correlations were found among the absolute values of Peak power in the GXT and the Maximum *(r=0.365, p=0.02)*, Minimum *(r=0.334, p=0.035)* and Average *(r=0.401, p=0.01)* power in the RAST. No relationships were found between the VO_2_max obtained from both aerobic tests and any performance indices in the RAST.

## Discussion

The main purpose of the present study was to examine if aerobic power influences repeated anaerobic exercise. The aerobic power was determined by a continuous aerobic test (GXT) performed under laboratory conditions. The protocol with the inclination manipulation was used in order to meet the maximal time requirement of the test, as mentioned in Material and Methods. In the event of speed manipulation only, some participants can be limited by their speed ability and cannot reach VO_2_max. The GXT is considered the “gold standard” in maximal oxygen uptake (VO_2_max) examination. The spiroergometry examination is quite expensive, however, and not commonly available for junior or adult non-professional soccer teams. On this account, indirect assessments of the VO_2_max are used in a daily routine. One of these field tests is the 20mPST (Léger & Lambert, 1982), which is relatively frequent in soccer and was therefore also chosen for the aerobic power determination in our study. The movement pattern of the 20mPST is more appropriate than GXT as to measure a component of fitness which is valid for a certain sport, the activity of that sport must be recreated as closely as possible (Wragg et al., 2000).

The repeated sprint ability was evaluated by RAST (Mackanzie, 2005), which is much more suitable for athletes where running forms the basis of the movement than the very popular and traditionally used Wingate anaerobic cycle test. The disadvantage of the RAST, as a test of RSA in soccer, can be an absence of multidirectional running (Wragg et al., 2000). Establishing relationships between fitness measures and match performance is problematic, however, given the random pattern of activity and the varying influence of tactics (Oliver et al., 2009).

The energy demands of a sport such as soccer are complex and difficult to quantify (Wragg et al., 2000). There is no doubt that the aerobic contribution to a single, short-duration sprint is relatively small. In addition, the metabolic recovery rate from single, high-intensity exercise is poorly predicted by VO_2_max. According to Cooke et al. (1997), the recovery rate differences in subjects with a similar VO_2_max imply that other factors influence recovery. Likewise, Bell et al. (1997) did not find any correlations between VO_2_max and the ability to recover after intermittent, high-intensity exercise.

The anaerobic ATP production is provided by considerable contributions from both PCr degradation and anaerobic glycolysis (Spencer et al., 2005). If the sprints are repeated for a particular period, e.g. 90 minutes of a soccer match, it is obvious that the anaerobic ATP production cannot be the sole energy source. The performance decline during the soccer game (Mohr et al., 2005) is related to the degradation and resynthesis rate of PCr (Spencer et al., 2005). The limited factor for PCr recovery is the availability of O2, even under normoxic conditions (Haseler et al., 1999). Consequently, the relative contribution of aerobic metabolism increases during the repeated high-intensive exercise with an insufficient recovery phase (Gaitanos et al., 1993; Bogdanis et al., 1996; Parolin et al., 1999; Spencer et al., 2005), which is specific to the movement pattern of field-based team sports. Furthermore, the ability to buffer H^+^ is a significant attribute for maintaining performance during brief, repeated sprints. Thus, the enhancing of aerobic fitness and muscle buffer capacity positively affects RSA improvement (Bishop et al., 2004). Apart from the level of aerobic power, individuals with faster VO_2_ kinetics during constant load exercise might also experience a faster adjustment of the VO_2_ during repeated sprint exercises leading to a shorter cumulated time and a lower relative decrease in speed (Dupont et al., 2005).

Nevertheless, in the present study, no relationship was found between maximal oxygen uptake and performance indices in repeated anaerobic exercise. The explanation can be in a modification of the RSA test protocol. This would mean that these results could be strictly specific to the employed RSA test. Balsom et al. (1992) have demonstrated that physiological and performance responses to repeated sprints are markedly influenced by the sprint distance while Meckel et al. (2009) showed that the aerobic system was more related to power maintenance in an intermittent activity with a high number of shorts repetitions (12 × 20 m) than to one with a low number of long repetitions (6 × 40 m). Therefore, the sprint number, sprint and recovery duration have to be considered as important factors which influence energy system contribution during repeated-sprint exercise. Some of the variables (maximal speed in a single sprint, total work done) can, however, be suggested to be general qualities of RSA independent of the RSA protocol used (Oliver et al., 2009).

The tested group of elite junior soccer players was highly homogenous, which can be another explanation of our results. Likewise, Bishop et al. (2003) summarized that the VO_2_peak was not a significant predictor of repeated sprint ability in a homogenous group of elite, female, team sport athletes.

There is also a question how the level of aerobic fitness (i.e. VO_2_max) influences repeated anaerobic exercise. The positive correlation between VO_2_max and RSA was found in studies with untrained or low to moderate trained subjects (Tomlin & Wenger, 2002; Bishop et al., 2004). According to Tomlin and Wenger (2002), caution should be used in extrapolating these results to highly trained individuals. The possibility of an aerobic fitness threshold exists, beyond which improvements in the VO_2_max do not translate into further enhancements of recovery. In the present study, all the participants were highly trained. Nevertheless, the results were sized according to the aerobic (VO_2_max, Peak power (W; W.kg^−1^)) or RAST indices and the best or worst 10 participants (unpresented data) were consequently correlated. No relationships were found between these aerobic and RSA indices. This procedure can be more beneficial for the aerobic fitness threshold searching in a more heterogeneous study group.

The various modifications of the 20mPST are considered a valid and reliable test for the prediction of VO_2_max (Léger & Lambert, 1982). The indirect measurement of VO_2_max, however, should be viewed with caution as the accuracy is about ± 15 % (Astrand et al., 2003). In the present study, a significant correlation between the spiroergometry results and the estimated VO_2_max values from the 20mPST was found.

The determination coefficient (*r^2^=0.146*) shows that approximately 85 % of this relationship is not clarified. There is also evidence of a significant mean bias (*p < 0.001*) when using 20mPST method to predict VO_2_max where the 20mPST method tends to underestimate GXT VO_2_max by between 3 and 6 ml.kg^−1^.min^−1^. According to the limits of agreement (Bland & Altman, 2010), the magnitude of the difference is excessively large. Some individuals could have a 20mPST VO_2_max value as high as 14 ml.kg^−1^.min^−1^ below GXT VO_2_max value, whereas some could return a 20mPST VO_2_max value of up to 5 ml.kg^−1^.min^−1^ higher than the GXT VO_2_max value. We can consequently conclude that the 20mPST does not provide a solid appraisement of VO_2_max. This result corresponds to Metaxas et al. (2005) who, in addition, consider the 20mPST an easy and helpful tool for coaches. The test result, however, should be preferably expressed as distance covered (endurance performance).

## Conclusions

The level of VO_2_max does not influence the performance indices in the repeated anaerobic exercise in elite junior soccer players. These results are strictly specific in relation to the RSA test used. The modification of the RSA test protocol or a more heterogeneous study group could possibly influence the results. Although, a relationship was found between the direct and indirect VO_2_max estimation, the VO_2_max values dispersion was described by approximately 15 % only. So, the estimation of VO_2_max in the 20mPST is too inaccurate and should not replace the laboratory spiroergometry examination. The 20mPST, however, might be an easily available test of endurance performance.

## Figures and Tables

**Table 1:** GXT and 20mPST results (N = 40)

Parameters	Mean ± s
**GXT**	
VO_2_max (ml.kg^−1^.min^−1^)	62.9 ± 5.12
Peak power (W)	453.5 ± 39.62
Peak power (W.kg^−1^)	6.4 ± 0.43
**20mPST**	
VO_2_max (ml.kg^−1^.min^−1^)	58.2 ± 2.86

s – standard deviation

**Table 2:** Paired t-test for 20mPST - GXT

	N	Mean	s	s_x^−^_
VO_2_max_20mPST (ml.kg^−1^.min^−1^)	40	58.19	2.86	0.45
VO_2_max_GXT (ml.kg^−1^.min^−1^)	40	62.92	5.12	0.81
Difference (ml.kg^−1^.min^−1^)	40	−4.73	4.81	0.76
95% CI for mean difference:	(−6.27, −3.19)			
t-test of mean difference = 0 (vs not = 0):	t-value = −6.21			
p-Value = 0.000				

N – number of participants, s – standard deviation, s_x_^−^ – standard error of the mean, CI – confidence interval

**Table 3:** RAST results (N = 40)

Parameters	Mean ± s
Total sprint time (s)	31.8 ± 1.10
Maximum power (W)	748.6 ± 85.46
Minimum power (W)	486.7 ± 68.94
Average power (W)	599.6 ± 66.25
Fatigue index (W.s^−1^)	8.2 ± 2.08

**Table 4:** Relationships among performance indices in the RAST, GXT and 20mPST

N = 40	**RAST**				
	TST (s)	Pmax (W)	Pmin (W)	Pavg (W)	FI (W.s^−1^)
**GXT**					
VO_2_max (ml.kg^−1^.min^−1^)	−0.086 (0.599)	−0.079 (0.626)	−0.056 (0.731)	−0.088 (0.589)	−0.036 (0.825)
Peak power (W)	0.045 (0.781)	0.365* (0.020)	0.334* (0.035)	0.401* (0.010)	0.109 (0.504)
Peak power (W.kg^−1^)	−0.236 (0.143)	−0.009 (0.956)	0.186 (0.250)	0.101 (0.534)	−0.182 (0.261)
**20mPST**					
VO_2_max (ml.kg^−1^.min^−1^)	−0.199 (0.219)	0.130 (0.426)	0.113 (0.488)	0.079 (0.626)	0.077 (0.638)

TST – total sprint time, Pmax – Maximum power, Pmin – Minimum power, Pavg – Average power, FI – Fatigue index.

The level of significance for each value is showed in brackets.

* Significant correlation at p < 0.05.
